# Thymic Epithelial Neoplasms: Focusing on the Epigenetic Alterations

**DOI:** 10.3390/ijms23074045

**Published:** 2022-04-06

**Authors:** Iason Psilopatis, Alexandros Pergaris, Kleio Vrettou, Stamatios Theocharis, Constantinos Troungos

**Affiliations:** 1 First Department of Pathology, Medical School, National and Kapodistrian University of Athens, 75 Mikras Asias Street, Bld 10, Goudi, 11527 Athens, Greece; iason.psilopatis@charite.de (I.P.); alexperg@yahoo.com (A.P.); kliovr1@gmail.com (K.V.); stamtheo@med.uoa.gr (S.T.); 2 Charité-University School of Medicine, Augustenburger Pl. 1, 13353 Berlin, Germany; 3 Department of Biological Chemistry, Medical School, National and Kapodistrian University of Athens, 75 Mikras Asias Street, Bld 16, Goudi, 11527 Athens, Greece

**Keywords:** thymoma, thymic carcinoma, TEN, epigenetics, non-coding RNA, DNA methylation, HDACI

## Abstract

Thymic Epithelial Neoplasms (TENs) represent the most common tumors of the thymus gland. Epigenetic alterations are generally involved in initiation and progression of various cancer entities. However, little is known about the role of epigenetic modifications in TENs. In order to identify relevant studies, a literature review was conducted using the MEDLINE and LIVIVO databases. The search terms thymoma, thymic carcinoma, thymic epithelial neoplasm, epigenetics, DNA methylation, HDAC and miRNA were employed and we were able to identify forty studies focused on TENs and published between 1997 and 2021. Aberrant epigenetic alterations seem to be involved in the tumorigenesis of thymomas and thymic carcinomas, with numerous studies reporting on non-coding RNA clusters and altered gene methylation as possible biomarkers in different types of TENs. Interestingly, Histone Deacetylase Inhibitors have shown potent antitumor effects in clinical trials, thus possibly representing effective epigenetic therapeutic agents in TENs. Additional studies in larger patient cohorts are, nevertheless, needed to verify the clinical utility and safety of novel epigenetic agents in the treatment of patients with TENs.

## 1. Introduction

The thymus is a secondary lymphoid gland located in the upper anterior mediastinum with its histologic architecture including the epithelium and the lymphoid component [1]. TENs represent a wide range of tumors deriving from the thymic epithelial cells that can be divided into thymomas and thymic carcinomas (TC). The World Health Organization (WHO) histopathological classification further subdivides thymomas into type A, type AB, type B1, type B2 and type B3 based on the morphology of epithelial tumor cells, the relative proportion of the lymphocytic component and the differentiation grade. TC are classified as type C TEN by the WHO [2,3,4].

Thymomas are considered as the most common neoplasms of the thymus gland, yet overall, thymomas are rare. The American Cancer Society estimates their incidence at 400 cases in the United States annually [5]. TCs account for 20% of all thymus cancers [5]. Based on patients diagnosed with thymus cancer between 2010 and 2016, the five-year relative survival rate is 71% for all surveillance, epidemiology, and end results (SEER) stages combined [6].

The majority of TEN are incidentally detected on chest X-ray or scan prior to symptom onset. Symptoms caused by thymus tumors either result from the compression of neighboring anatomical structures such as the airways and the superior vena cava, or are paraneoplastic, with myasthenia gravis (MG) representing the most common autoimmune disease associated with TEN [7].

Diagnostic evaluation of TEN includes, in addition to a physical examination, a computed tomography (CT) scan of the chest, usually combined with a positron emission tomography (PET) scan. magnetic resonance imaging (MRI) may be used to measure the tumor’s size. Definite diagnosis requires biopsy of the tumor mass [8]. Of note, the thymus gland physiologically disappears after puberty. As such, when children or infants present with thymic pathologies, the common diagnosis techniques like X-ray, CT or scintigraphy may not be always used, given their dangerous repercussions for future quality of life. Interestingly, when it comes to children, the roles of diet and especially obesity seems to play an important role in the altered morphology and function of TEN cells due to the infiltration and accumulation of adipocytes in tumor-bearing thymuses [9].

For patients with early-stage resectable TEN, thymectomy represents the first-line therapy. Patients with more advanced stage TEN may also be treated with radiation and/or chemotherapy after surgery, especially in cases of incomplete tumor resection [10].

**Figure 1:**
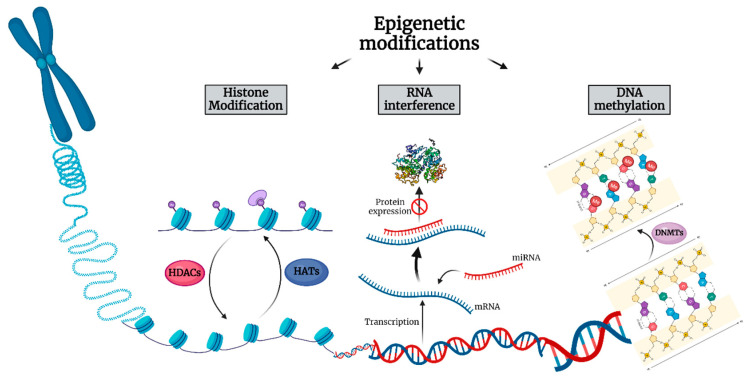
The three main mechanisms of epigenetic alterations comprised of histone acetylation, protein expression obstruction through RNA interference and DNA methylation. HDAC: Histone deacetylase, HAT: Histone acetyltransferase, DNMT: DNA methyltransferase.

Cancer initiation and progression is now realized to involve genetic alterations along with epigenetic abnormalities. Epigenetics refers to the study of heritable changes in gene expression that enable cells to have distinct identities by regulating what genetic information can be accessed by cellular machinery without changing the primary DNA sequence [11]. Epigenetic modifications include DNA methylation, posttranslational modifications of histone proteins, nucleosome remodeling and non-coding RNAs, specifically microRNA (miRNA) expression (Figure 1) [12].

DNA methylation is regulated by a family of DNA methyltransferases (DNMTs) and involves the transfer of a methyl group onto the fifth carbon position of the cytosine to form 5-methylcytosine [13]. This heritable epigenetic mechanism regulates gene expression by recruiting proteins involved in gene repression or by inhibiting the binding of transcription factor(s) to DNA [14]. In cancer cells, global hypomethylation of the cancer genome, promoter hypermethylation of tumor suppressor genes and potentially direct mutagenesis of 5-methylcytosine-containing sequences through deamination of methylated cytosine can promote cancer initiation and evolution [15].

Post-translational modification of histone proteins by acetylation reduces the affinity of histones for the negatively charged DNA, thus enabling DNA strands to uncoil and transcription to occur [16]. The level of histone acetylation relies on the opposing actions of histone acetylases (HATs) and histone deacetylases (HDACs) [17]. HDACs catalyze the removal of acetyl groups on the NH2-terminal lysine residues of core nucleosomal histones, which generally results in transcriptional repression and the silencing of tumor-suppressor genes [18]. As a consequence, deregulation of histone acetylation can promote the development of human cancer, as shown by a great number of researchers who focused on revealing the link between histone acetylation/deacetylation and tumorigenesis [19,20,21].

The majority of the human genome is transcribed into non-coding RNAs [22]. miRNAs and long non-coding RNAs (lncRNAs) represent two major families of these non-protein-coding transcripts that can regulate fundamental cellular processes [23]. Dysregulation of miRNAs and lncRNAs is critical to cancer development and progression. Both miRNAs and lncRNAs have been reported to act as either oncogenes or tumor suppressors to regulate cancer biology via diverse molecular mechanisms, such as amplification, deletion, abnormal epigenetic or transcriptional regulation [24]. miRNAs are in particular associated with the hallmarks of neoplasia, ranging from angiogenesis induction to apoptosis resistance and metastasis activation [23].

In this review, we extensively investigated epigenetic alternations in TEN, incorporating research works that explored their role in carcinogenesis in both in vitro and in vivo studies. In particular, we searched for possible epigenetic biomarkers in TEN, as well as possible treatment options. The literature review was conducted using the MEDLINE and LIVIVO databases. The search terms *thymoma, thymic carcinoma, thymic epithelial neoplasm, epigenetics, DNA methylation, HDAC* and *miRNA* were employed and we were able to identify forty studies focused on TEN and published between 1997 and 2021.

## 2. Alterations of Non-Coding RNAs in Thymic Epithelial Neoplasms

Alterations of non-coding RNAs play an established role in TEN development and progression. A large number of studies have assessed the epigenetic abnormalities associated with alterations of non-coding RNAs in TEN patient tissue samples (Table 1). Ganci et al. performed miRNA expression profiling by microarray analysis of formalin-fixed paraffin embedded tissue in TENs versus normal thymic tissues and identified 87 miRNAs differently expressed between thymic tumor and normal samples, with selected miRNAs distinguishing the different TEN histotypes. miRNA-21-5p was found to be up- and miRNA-145-5p down-regulated in TEN, whereas miRNA-142-5p, miRNA-363-3p and miRNA-16-2-3p were shown to be predicted to target molecular pathways involved in thymic carcinogenesis, such as Baculoviral IAP Repeat Containing 3 (BIRC3), SCYA20 and MYC [25]. Wei et al., found miRNA-140, miRNA-450b, miRNA-542, miRNA-639, miRNA-3613 and miRNA-3913–1 to be positively associated and miRNA-1976 to be negatively associated with overall survival in thymomas. In terms of disease-free survival, let-7a-1, let-7a-2 and let-7a-3 miRNAs positively correlated with disease-free survival in thyomas, whereas miRNA-324 negatively correlated with it. Binary tree prediction discriminated TC from type A-B3 thymomas by identifying 91 miRNAs with differential expression between TC and non-type C thymomas [26]. Bellissimo et al. selected miRNA-21-5p, miRNA-148a-3p, miRNA-141-3p, miRNA-34b-5p, miRNA-34c-5p, and miRNA-455-5p to evaluate their expression in blood plasma and peripheral blood mononuclear cells and confirmed increased levels for miRNA-21-5p and miRNA-148a-3p. Plasma levels of miRNA-21-5p and miRNA-148a-3p were significantly reduced during follow-up after thymectomy [27]. In their study, Radovich et al. revealed 100% concordance between gene expression clusters and TEN histotype. A large miRNA cluster on chromosome 19q13.42 (C19MC) was shown to be significantly overexpressed in all A and AB TEN and virtually absent in the other thymomas and normal tissues, while overexpression of C19MC activated the oncogenic PI3K/AKT/mTOR pathway [28]. Enkner et al. suggested, on the other hand, that C19MC expression is silenced in TCs as a result of promoter methylation, and that the expression of miRNA cluster C14MC on chromosome 14q32 is decreased in TCs as compared to type A thymomas. Moreover, miRNA-21, miRNA-9-3 and miRNA-375 were up-, whereas miRNA-34b, miRNA-34c, miRNA-130a and miRNA-195 were down-regulated in TCs [29]. Ji et al. performed RNA sequence analysis and detected 65 differentially-expressed lncRNAs in thymomas, including AFAP1-AS1, LINC00324, ADAMTS9-AS1, VLDLR-AS1, LINC00968, and NEAT1 that have been validated with The Cancer Genome Atlas (TCGA) database. 1695 miRNAs were also reported to be overexpressed in thymomas. A network analysis of the lncRNA-mRNA-miRNA regulation axes identified a cluster of miRNAs upregulated in thymomas, triggering the expression of target protein-coding genes and disrupting various biological pathways, such as the PI3K/Akt/mTOR, FoxO, and Hypoxia-Inducible Factor (HIF)-1 signaling pathways [30]. Furthermore, the lncRNAs ADAMTS9-AS1, HSD52, LINC00968 and LINC01697 were described as potential markers in terms of accurate patient stratification in low versus high risk TEN, as well as in effective recurrence probability prediction [31]. Gene expression analysis by microarray in a cohort of fresh frozen TEN and normal tissues identified LINC00174 as up-regulated in TEN cases, with its expression positively correlating with a 5-genes signature. LINC00174 favored the expression of Syntabulin (SYBU), FEM1B, and Stearoyl-CoA Desaturase 5 (SCD5) genes by sponging miRNA-145-5p, thus affecting lipid metabolism and, consequently, TEN cell migration [32]. In their study, Wang et al., found LOXL1-AS1 and HSPA9 to be overexpressed in TEN and associated with poor prognosis. miR-525-5p expression was down-regulated, predicted patient prognosis and inhibited the expression of HSPA9 protein by targeting the 3′-untranslated region (UTR) of HSPA9 mRNA. LOXL1-AS1 promoted HSPA9 expression as a sponge targeting miR-525-5p. An in vivo tumor-burdened assay also showed that knockdown of miR-525-5p promoted tumorigenesis by stimulating the expression of HSPA9 [33]. In thymomatous MG, the aberrant decrease of miRNA-19b regulated thymic stromal lymphopoietin (TSLP) expression and contributed to T helper type 17 cells development [34]. Similarly, miRNA-20b was found to be down-regulated in patients with thymoma-associated MG and to target Nuclear Factor of Activated T-cells 5 (NFAT5) and Calmodulin Binding Transcription Activator 1 (CAMTA1), thus inhibiting T-cell proliferation and activation. Of note, the expression levels of miRNA-20b and NFAT5/CAMTA1 were inversely correlated in patients with thymomatous MG [35]. Yang et al., reported on the disparate role of the pseudogene RP11-424C20.2 and its parental gene UHRF1, and showed that high expression levels predicted better overall survival in TEN through the regulation of tumor-infiltrating immune cell levels, which is mediated at least partly through Interferon (IFN)-γ-mediated Clathrin Light Chain A (CLTA)-4 and Programmed Death-Ligand 1 (PD-L1) pathways. miRNA-378a-3p was down-, whereas miRNA-422a up-regulated in thymomas [36]. Analyses of 87 thymoma samples with complete MG information revealed distinct expression of immune-related genes and lncRNAs in thymoma with and without MG. The study found the methylation level of the immune-related lncRNAs AC004943.1, WT1-AS and FOXG1-AS1 to be significantly decreased in TEN and to correlate with their expression. Gene Ontology and Kyoto Encyclopedia of Genes and Genomes analyses revealed the functional pathways of the target genes of these immune-related lncRNAs with transcription coregulator activity and cell cycle representing the most enriched pathways for targets of lncRNA AC004943.1, and actin binding and axon guidance the most enriched pathways for targets of lncRNA WT1-AS, respectively [37]. Chen et al., described that the three lncRNAs: LINC00665, NR2F1-AS1 and RP11-285A1.1, as well as the four miRNAs hsa-miRNA-143, hsa-miRNA-141, hsa-miRNA-140 and hsa-miRNA-3199, were significantly associated with prognosis and overall survival of TEN [38].

## 3. Alterations of DNA Methylation in TENs

Numerous studies have investigated the effects of DNA methylation alterations in TEN (Table 2). Bioinformatic analysis of the TCGA dataset revealed 5155 and 6967 hyper- and hypo-methylated CpG sites in type A–B3 TEN and TC, respectively, of which 3600 were located within the gene promoter regions. One hundred and thirty-four genes were found to be silenced by promoter hypermethylation, while 174 mRNAs were up-regulated. Cox regression analysis showed significant association between the methylation levels of 187 sites and the overall survival in patients with TEN, with cg05784862 (KSR1), cg07154254 (ELF3), cg02543462 (ILRN), and cg06288355 (RAG1) representing independent prognostic factors [39]. Bi et al., attempted to examine the whole-genome DNA methylation status of TEN and identify differences in thymoma DNA methylation profiles. More than 10,000 CpGs were found to be differentially methylated between thymoma types A and B, with 36 genes having differentially methylated CpG sites in their promoter region. A Kyoto Encyclopedia of Genes and Genomes analysis identified focal adhesion and regulation of the actin cytoskeleton as the most enriched pathway of differentially methylated genes between tumors and healthy controls. Among the 29 genes that were hypomethylated with a high expression, zinc finger protein 396 and Fraser extracellular matrix complex subunit 1 had the largest area under the curve [40]. Another study, investigating whether a common polymorphism (×579G>T: rs1569686) in the promoter of the DNMT3B gene increases the risk to develop thymomatous MG, found a statistically significant association of the DNMT3B-579T allele and the TT homozygous genotype with the risk of TEN [41]. Coppedè et al. investigated MutL homolog 1 (MLH1), O(6)-methylguanine-DNA methyltransferase (MGMT), Cyclin Dependent Kinase Inhibitor (CDKN2A) and Ras association domain family 1 isoform A (RASSF1A) methylation levels in blood and tumor specimens from 69 patients with thymoma-associated MG and in the adjacent normal thymus available from 44 of them, and concluded that promoter methylation levels of these genes were neither increased in thymoma nor associated with the histopathological features of TEN [42]. On the contrary, Mokhtar et al. found MGMT methylation to be significantly more frequent in advanced TEN than in early thymomas [43]. Suzuki et al., also reported a significantly higher methylation index in TC than in thymomas, with the Secreted Protein Acidic and Rich in Cysteine (SPARC) genes being methylated in all examined TC samples [44]. The analysis of global methylation levels and of the promoter methylation status of the tumor suppressor genes (TSG) hMLH1, MGMT, p-16^INK4a^, RASSF1A, Fragile Histidine Triad Diadenosine Triphosphatase (FHIT), Anaphase-promoting complex subunit 1 (APC1A), Retinoic acid receptor β (RARB), Death-associated protein kinase (DAPK) and E-cadherin in 65 TEN samples revealed hypermethylation and decreased TSG expression in types B1 or higher thymomas. In comparison with early-stage TEN, global DNA methylation levels were found to be reduced, whereas DNMT1, DNMT3a, and DNMT3b expression was increased in advanced-stage thymomas [45]. The methylation status determination in a series of 13 resected thymoma samples by the sodium bisulfite-modification method followed by sequencing analysis revealed a significantly high frequency of methylation in the F-box and WD40 domain protein 7 (FBXW7) β-form promoter in types B1 or higher TEN [46]. Hirabayashi et al., examined 36 thymomas and four TC, and observed hypermethylation of the promoter region in the CDKN2 gene in four thymomas and one TC using a PCR-based assay, as well as genomic Southern hybridization [47]. Another relevant study examining aberrant DNA methylation of DAPK, p-16, MGMT and Hyperpigmentation, Progressive, 1 (HPP1) genes in 26 thymomas and 6 TC described the frequency of aberrant DNA methylation to correlate with the histopathologic TEN type and to be, consequently, higher in TC than thymoma [48]. Furthermore, Iaiza et al. found that the methyltransferase METTL3 is overexpressed in TEN and responsible for the induction of c-MYC expression through methylation and delocalization of the lncRNA MALAT1. Silencing of METTL3 combined with cisplatin or c-MYC inhibitor promotes apoptosis in TEN cells [49]. Bisulfite pyrosequencing in 46 TEN and 20 paired thymus tissues revealed higher promoter methylation of G protein subunit gamma 4 (GNG4), growth hormone secretagogue receptor (GHSR), homeobox D9 (HOXD9) and spalt like transcription factor 3 (SALL3) in TC than in thymoma. Higher promoter methylation of these four genes correlated with a significantly worse relapse-free survival in all TEN types [50]. In a subsequent study, the same study group speculated that DNA methylation of GHSR correlates with a shift from native to variant expression, thus inducing the tumorigenesis of thymoma, yet not TC [51]. Coppedè et al. investigated GHSR methylation levels in blood, tumor tissue, and adjacent healthy thymic tissue from 65 MG patients and observed hypermethylation in all WHO histological subtypes, but particularly at advanced disease stages [52]. The AutoImmune REgulator (AIRE) promoter was found to be hypomethylated in AIRE-negative TEN with variable individual patterns, thus indicating that the lack of expression does not result from the CpG-methylation mediated silencing of the AIRE gene promoter [53]. Ye et al., employed bisulphite sequencing to identify hypomethylation in the 5′ promoter region of the pro-opiomelanocortin (POMC) gene in five TC tumors resected from patients with ectopic adrenocorticotropic hormone (ACTH) syndrome and concluded that hypomethylation of this promoter correlates with POMC overexpression and the ectopic ACTH syndrome in TC [54]. The methylation-sensitive high-resolution melting technique for the analysis of DNA methylation levels of genes involved in one-carbon metabolism and DNA methylation in blood, tumor tissue, and healthy thymic epithelial cells from thymomatous MG patients showed significantly higher methylation of the methylenetetrahydrofolate reductase (MTHFR) promoter in thymomas, as well as some degrees of methylation of the DNMT3A gene in thymic tissue with respect to blood [55]. Masunaga et al., observed no methylation in the Phosphatase and Tensin homolog (PTEN) promoter region in TEN tumor cells [56]. Moreover, Deleted in liver cancer one (Dlc1) isoform 2 was described to undergo selective hypermethylation in oncogenic Kirsten rat sarcoma virus oncogene homolog (K-Ras) 2 induced TEN and significantly decrease the overall survival in Dlc1 gene trap mice [57]. Saito et al., showed that TC with Tet Methylcytosine Dioxygenase 2 (TET2) mutations had more hypermethylated genes and that hypermethylation correlated with down-regulation of gene expression [58]. Another study, examining DNA methylation by bisulfite pyrosequencing, revealed significantly higher methylation levels of glutamate decarboxylase 1 gene (GAD1) in TC tissues, with GAD1 methylation exhibiting high sensitivity and specificity for discriminating between TC and thymoma. More importantly, patients with TEN with high GAD1 DNA hypermethylation showed significantly shorter relapse-free survival rates [59]. Interestingly, the positive correlation between promoter DNA methylation and GAD1 expression in TEN was attributed to the inhibition of a Polycomb repressive complex 2 (PRC2) by the methylation of CCCTC-binding factor (CTCF) −3, with H3K27me3 levels markedly reduced at the GAD1 promoter. Through multiomic analysis of the TCGA, Yang et al. reported that KIT proto-oncogene ligand (KITLG) represents a new hallmark of type A and AB thymomas by inducing aberrant alterations of mRNA, miRNA and DNA methylation [60].

## 4. Alterations of Histone Modifications in Thymic Epithelial Neoplasms

Several studies have examined the role of histone modification alterations in TEN by mainly focusing on modification of HDAC activity (Table 3). Arjonen et al., performed image-based high content drug screening to assess tumor cell specific responses and identified selective sensitivity of the TEN cells to HDAC Inhibitors (HDACI) Belinostat and Panobinostat [61]. A phase II study of Belinostat in 41 patients with recurrent or refractory advanced TEN demonstrated partial responses in two patients with thymoma, but no responses among patients with TC, with survival of patients with thymoma being significantly longer than that of patients with TC [62]. A phase I/II trial of Belinostat in combination with cisplatin, doxorubicin and cyclophosphamide in TEN, on the other hand, revealed objective response rates of 64% in thymomas and 21% in TC, respectively [63]. Bellissimo et al. found that treatment of TC1889 cells with the HDAC inhibitor Valproic Acid (VPA) led to miRNA-145-5p up-regulation and exhibited antitumor effects, such as reduction of cell viability, colony forming ability and migration capability. Interestingly, VPA also seemed to enhance the response of TEN cells to chemotherapy [64].

## 5. Conclusions

The present review describes epigenetic alterations, searches for possible epigenetic biomarkers and explores possible novel treatment options in TEN. Alterations of non-coding RNAs seem to play an important role in TEN carcinogenesis and progression with numerous studies identifying up- or down-regulation of different miRNA/lncRNA clusters in thymomas and TC. Selected non-coding RNAs may distinguish different TEN histotypes, given their differential expression in normal thymi, thymomas and TC, target various molecular oncogenic pathways involved in TEN pathophysiology, such as the PI3K/AKT/mTOR, the FoxO, or the HIF-1 signaling pathway, as well as regulate immune cell proliferation and activation in thymomatous MG. Moreover, up- or down-regulation of non-coding RNAs either positively or negatively correlates with overall and disease specific survival in TEN, depending on each miRNA/lncRNA cluster. In terms of DNA methylation alterations, multiple CpG sites seem to be either hyper- or hypo-methylated in different histopathologic TEN types. Many of these CpG sites are located within the gene promoter regions, with DNA methylation alterations thus resulting in either the activation or silencing of important tumor suppressor and DNA repair genes associated with TEN tumorigenesis. Notably, MG-associated thymomas seem to represent a molecularly distinct subtype of TEN, given that hypermethylation of certain genes such as MLH1, MGMT, CDKN2A, and RASSF1A might be more frequent in TC than in thymomas [43,45,47,48], yet hypermethylation of these genes is neither frequent in thymomatous MG tissues nor does it correlate with the histopathologic features of the tumor [42]. Importantly, DNA methylation levels seem to significantly correlate with overall and relapse-free survival in patients with TEN, thus representing a significant prognostic factor. All in all, both non-coding RNAs and DNA methylation levels could be regarded as potential epigenetic biomarkers in terms of accurate patient stratification in low versus high risk TEN, effective recurrence probability prediction and, most importantly, overall patient survival, with a great number of studies incorporating hundreds of samples from datasets, such as the TCGA, and providing reliable and reproducible data that prove the role of epigenetic modifications in TEN pathogenesis and prognosis [31,36,39,60]. Interestingly, HDACIs seem to represent promising epigenetic therapeutic agents with potent antitumor effects in TEN. Belinostat has already been tested in a phase II clinical trial, showing promising results in a small patient collective with recurrent or refractory advanced thymomas [62], thus opening the way for further clinical studies to be conducted in larger patient collectives, with an aim to verify the clinical utility and safety of epigenetic modulators and develop novel treatments targeting different epigenetic alterations in TEN.

## Figures and Tables

**Table 1:** Alterations of non-coding RNAs in TENs.

Non-Coding RNA	Cell Lines/Tissue Samples	Methods	Main Results	References
87 miRNAs	54 thymic tumor samples, 12 normal counterparts	Reverse transcription quantitative real-time Polymerase Chain Reaction (RT-qPCR), Immunohistochemistry	Statistically significant differential expression of miRNAs between different histotypes of thymomas and TC. Up-regulation of miRNA-21-5p and down-regulation of miRNA-145-5p in TEN. BIRC3, SCYA20 and MYC as putative targets of miRNA-142-5p, miRNA-363-3p and miRNA-16-2-3p in TC samples.	[25]
91 miRNAs	17 type A, 38 type AB, 13 type B1, 31 type B2, 13 type B3, 11 type C thymomas	KEGG pathway analysis of miRNA target genes	miRNA-140, miRNA-450b, miRNA-542, miRNA-639, miRNA-3613 and miRNA-3913–1 positively, miRNA-1976 negatively correlated with overall survival. let-7a-1, let-7a-2 and let-7a-3 positively, miRNA-324 negatively correlated with disease-free survival. let-7a-1, let-7a-2, let-7a-3, miRNA-140, miRNA-324, miRNA-639 and miRNA-3613 down-regulated in TC patients.	[26]
miRNA-21-5p, miRNA-148a-3p, miRNA-141-3p, miRNA-34b-5p, miRNA-34c-5p, miRNA-455-5p	Peripheral blood samples from five patients with TEN	MicroRNA expression analysis	miRNA-21-5p and miRNA-148a-3p significantly up-regulated in blood plasma from TEN patients at the time of surgery and significantly reduced during follow-up.	[27]
Large microRNA cluster on chr19q13.42	4 type A, 2 type AB, 1 type B2, 5 type B3, 1 type C thymomas, 3 normal tissues, validation set of 35 thymic malignancies, IU-TAB1 cell line	RNA-sequencing, qPCR, protein analyses, drug sensitivity experiments	100% concordance between gene expression clusters and TEN histotype. Significant overexpression of the miRNA cluster on chr19q13.42 in all type A and AB thymomas. miRNA cluster overexpression activates the PI3K/AKT/mTOR pathway. Treatment of the IU-TAB1 cell line with PI3K/AKT/mTOR inhibitors markedly reduced cell viability.	[28]
miRNA cluster C14MC on chromosome 14q32, miRNA-21, miRNA-9-3, miRNA-375, miRNA-34b, miRNA-34c, miRNA-130a, miRNA-195	18 type A, 19 type B3 thymomas, 35 TC	Cancer gene panel sequencing, miRNA sequencing, fluorescence in situ hybridization (FISH), immunohistochemistry	C19MC miRNA cluster highly expressed in type A thymomas, but completely down-regulated in TC. miRNA cluster C14MC down-regulated in TC. Up-regulation of miRNA-21, miRNA-9-3 and miRNA-375 in TC. Down-regulation of miRNA-34b, miRNA-34c, miRNA-130a and miRNA-195 in TC. Increased PDGFRA in TC and PD-L1 in type B3 TEN and TC.	[29]
65 differentially-expressed lncRNAs, 1695 miRNAs	25 thymomas, 25 healthy thymus specimens	lncRNA-miRNA-mRNA functional enrichment analyses	65 differentially-expressed lncRNAs in thymomas, including AFAP1-AS1, LINC00324, ADAMTS9-AS1, VLDLR-AS1, LINC00968, and NEAT1. 1695 miRNAs overexpressed in thymomas. Cluster of miRNAs up-regulated in thymomas, with disruption of the PI3K-Akt, FoxO, and HIF-1 signaling pathways.	[30]
lncRNAs ADAMTS9-AS1, HSD52, LINC00968, LINC01697	TCGA	Statistics	ADAMTS9-AS1, HSD52, LINC00968 and LINC01697 effectively divide patients into high and low risk subgroups. lncRNAs classifier as an independent recurrence risk factor predictor with a larger net benefit than the Masaoka staging system.	[31]
lncRNA LINC00174	Four thymomas of different histotypes, 2 thymi TC1889 cell line	Bioinformatics analysis of expression data from TCGA and Istituto Regina Elena thymoma cohorts, cDNA reverse transcriptase, RT-q PCR	LINC00174 up-regulated and positively correlated with a 5-genes signature in TEN. LINC00174 and its associated 5-genes signature prognostic in TEN. LINC00174 favors the expression of SYBU, FEM1B, and SCD5 genes by sponging miRNA-145-5p. LINC00174-associated gene SCD5 impacts on cell migration and lipid metabolism.	[32]
miR-525-5p	42 thymoma, 28 TC, 30 normal thyroid tissue samples Thy0517, Ty-82, HBT8810	RT-qPCR, dual luciferase reporter assay, cell counting kit 8 assay, flow cytometry, transwell assay, western blot analysis, tumor-burdened assay	LOXL1-AS1 and HSPA9 overexpressed and associated with poor prognosis in thymoma and TC. Down-regulation of miRNA-525-5p expression correlated with poor prognosis. miRNA-525-5p down-regulated the expression of HSPA9 protein by targeting the 3’-untranslated region of HSPA9 mRNA. LOXL1-AS1 up-regulated the expression of HSPA9 as a sponge targeting miRNA-525-5p. Knockdown of miRNA-525-5p promoted the expression of HSPA9 in animal experiment results.	[33]
miRNA-19b	nine type A, 11 type B1, 20 type B2, 12 type B3 thymomas, 11 normal thymi	Luciferase reporter assay, qRT-PCR, western blot	TSLP significantly decreased in thymomas. TSLP post-trancriptionally regulated by miRNA-19b. Negative association of miRNA-19b with the expression of TSLP mRNA and protein in TENs.	[34]
miRNA-20b	Thymoma tissue specimens HEK293T cell line	qRT-PCR, MTT assay, cell cycle analysis, immunostaining, flow cytometric analysis, western blot	miRNA-20b expression down-regulated in thymoma tissue specimens and serum from patients with thymoma-associated MG. T cell proliferation and activation inhibited by ectopic overexpression of miRNA-20b. miRNA-20b targeted NFAT5 and CAMTA1, and inhibited their expression in cultured cells. Expression levels of miRNA-20b and NFAT5/CAMTA1 inversely correlated in patients with thymomatous MG.	[35]
RP11-424C20.2, miRNA-378a-3p, miRNA-422a	DreamBase, TCGA	RP11-424C20.2 cellular localization prediction, GO and KEGG enrichment analysis, correlation of UHRF1 expression with immune infiltration analysis	Pseudogene RP11-424C20.2 and its parental gene UHRF1 frequently up-regulated and positively correlated in TEN. RP11-424C20.2 as a competing endogenous RNA (ceRNA) increased UHRF1 expression through sponging miRNA-378a-3p. Strong correlation of UHRF1 with immune-related biological processes. UHRF1 expression significantly associated with immune infiltration. RP11-424C20.2/UHRF1 axis regulated TEN immune escape through IFN-γ-mediated CLTA-4 and PD-L1 pathway.	[36]
lncRNAs AC004943.1, WT1-AS, FOXG1-AS1	11 type A, 26 type AB, 10 type B1, 22 type B2, nine type B3 thymomas, 9 TC	Statistical analysis	205 mRNAs and 56 lncRNAs up-regulated in thymomatous MG. 458 mRNAs and 84 lncRNAs down-regulated in thymoma with MG patients. Methylation level of lncRNAs AC004943.1, WT1-AS and FOXG1-AS1 was significantly decreased in TEN tissues and correlated with their expression. Targets of the immune-related lncRNA FOXG1-AS1 enriched in small GTPase binding and herpes simplex virus 1 infection. lncRNA AC004943.1 mostly targets transcription coregulator activity and cell cycle pathways. lncRNA WT1-AS targets most enriched in actin binding and axon guidance.	[37]
lncRNAs LINC00665, NR2F1-AS, RP11-285A1.1, miRNAs hsa-miRNA-143, hsa-miRNA-141, hsa-miRNA-140, hsa-miRNA-3199	16 samples of type A, 35 samples of type AB, 57 samples of type B thymomas, 11 samples of TC, two samples of normal tissue.	GO and KEGG pathway annotation of DEmRNAs, overall survival analysis and establishment of the TEN-specific prognostic significance	Possible binding of lncRNA LINC00665 with mRNAs MYO10 and WASF3 through miRNAs hsa-miRNA-140 and hsa-miRNA-3199. Indirect interaction of lncRNA NR2F1-AS1 with the mRNAs FBN1, GALNT16, HAND2, and MCAM via hsa-miRNA- 140, hsa-miRNA-139, and hsa-miRNA-141. DOCK11, MCAM, MYO10, and WASF, which contained lncRNA LINC00665, lncRNA NR2F1-AS1, and lncRNA RP11-285A1.1, and hsa-miRNA-143, hsa-miRNA-141, hsa-miRNA-140, and hsa-miRNA-3199, were closely related to the prognosis and overall survival in TEN.	[38]

**Table 2:** Alterations of DNA methylation in TENs.

DNA Methylation Sites	Cell Lines/Tissue Samples	Methods	Main Results	References
5155 hyper-,6967 hypo-methylated CpG sites in type A–B3 TEN and TC	TCGA thymoma datasets with DNA methylation profiles of 124 tumor tissues and 2 matched adjacent normal tissues from 124 cases of thymoma, 54 thymomas, 46 TC	Statistical analysis, pyrosequencing	5155 and 6967 hyper- and hypomethylated CpG sites in type A–B3 and type C group, with 3600 located within the gene promoter regions, 134 genes silenced by promoter hypermethylation and 174 mRNAs up-regulated. Significant correlation between the methylation levels of 187 sites and overall survival in patients with TEN. KSR1, ELF3, ILRN, and RAG1 identified as independent prognostic factors for overall survival in TEN after adjusting for Masaoka staging.	[39]
10,000 CpG sites	1 atypical type A, 1 type A, 1 type AB, 1 type B1, 1 type B2, 2 type B3 thymomas, 1 atypical TC	DNA isolation and bisulfite treatment, illumina 850K methylation microarray, statistical analysis	3998 hyper-, 6016 hypomethylated differentially methylated CpGs between type A and B thymoma. ICAM3, APBB1IP, IFI16, PARVG, CCM2, INPP5D and SP110 correlated with Fc gamma R-mediated phagocytosis, Fc epsilon RI signaling pathway, cell adhesion molecules and focal adhesion. FEZ2, PTPRE, ATP2A2, CBLB, C5orf45, CPE, FSTL1, ZNF396, FRAS1, NAV2 and LCA5 as potential diagnostic biomarkers for type A and B thymomas.	[40]
DNMT3B-579T allele	Peripheral blood of 324 AChR+ MG patients, 735 healthy matched unrelated controls, 94 patients with thymoma	Genotyping analysis performed by means of PCR-RFLP techniques	Statistically significant association of the DNMT3B -579T allele and the TT homozygous genotype with TEN risk.	[41]
MLH1, MGMT, CDKN2A, RASSF1A	Blood samples and surgically resected thymomas from 69 patients with thymomatous MG and in the adjacent normal thymus available from 44 of them	Methylation sensitive-high resolution melting	No correlation between promoter methylation levels of MLH1, MGMT, CDKN2A, and RASSF1A genes and histopathological features of TEN or MG symptoms.	[42]
MGMT	four type A, 12 type AB, 13 type B1, 7 type B2, eight type B3 thymomas, 23 TC	Nested methylation-specific PCR (MSP), immunohistochemical analysis	Significant correlation between MGMT methylation and loss of its protein expression. MGMT methylation and loss of protein expression significantly more frequent in TC than in early thymomas.	[43]
SPARC	six thymoma, five TC, 22 non-small cell lung carcinoma samples	MSP, mutation assay	Elevation of tumor markers and higher methylation index significantly more frequent in TC than in thymomas. Patients with TC showed poorer prognosis than thymoma patients. Absent EGFR, human epidermal growth factor receptor 2 (HER2), or K-Ras mutations in TC or thymomas examined.	[44]
hMLH1, MGMT, p-16^INK4a^, RASSF1A, FHIT, APC1A, RARB, DAPK, E-cadherin	10 type A, 10 type AB, 12 type B1, nine type B2, seven type B3 thymomas, 17 TC	Real-time RT-PCR, nested MSP, immunohistologic analysis of global DNA methylation, measurement of global DNA methylation by an ELISA-like reaction	Promoters of hMLH1, MGMT, p-16^INK4a^, RASSF1A, FHIT, APC1A, RARB, DAPK and E-cadherin hypermethylated in thymomas, with histologic features associated with poor patient prognoses. Methylation detected in at least one promoter region of almost all types B2/B3/C thymomas. Expression of hMLH1, MGMT, FHIT, APC1A, RARβ, and E-cadherin down-regulated in more advanced TEN. Significant correlations between global hypomethylation and WHO histologic criteria. DNMT1, DNMT3a, DNMT3b genes overexpressed in TEN. Negative correlation between DNMT3b levels and global DNA methylation content.	[45]
FBXW7 β-form promoter	one type A, three type AB, five type B1, three type B2, one type B3 thymomas	Genomic sodium bisulfite sequencing analysis	Methylation status is significantly associated with histological classification.	[46]
CDKN2	36 thymomas (non-invasive type, 16 cases; invasive type, 20 cases), three TC	Immunohistochemistry, PCR-SSCP, sequencing, PCR-based methylation assay, southern hybridization	No mutation of p53 and CDKN2 genes detected in the thymomas and TC examined. Polymorphism in the 3′ untranslated region of exon 3 of CDKN2 detected in 5 thymoma cases. Hypermethylation in the promoter region of CDKN2 in four thymomas and one TC.	[47]
DAPK, p-16, MGMT, HPP1	one type A, six type AB, 10 type B1, four type B2, five type B3 thymomas, six TC	Sodium bisulfite modification	Aberrant methylation more frequent in TC than in thymoma. Frequency of tumors with methylation of multiple genes higher in advanced thymomas.	[48]
METTL3	two type A, five type AB, three type B1, six type B2, one type B3 thymomas, five TC samples TC1889 cell line	Immunohistochemistry, clonogenic assays, real-time qRT-PCR analysis, lysis and immunoblotting analysis, polysome profiling and treatment with puromycin, m6A immunoprecipitation, FISH	METTL3 overexpressed in TEN. Silencing of METTL3 expression in TC cells resulted in reduced cell proliferation and overall translation rate. METTL3 responsible for the induction of c-MYC expression in TEN cells. High expression of c-MYC protein enabled by lncRNA MALAT1, which is methylated and delocalized by METTL3. JQ1 inhibitor inhibits proliferation and induces apoptosis.	[49]
GNG4, GHSR, HOXD9, SALL3	five type A, two type AB, four type B1, 10 type B2, and nine type B3 thymomas, 12 TC, four NECTT samples	Bisulfite conversion of genomic DNA, bisulfite pyrosequencing	92 CpG islands significantly hypermethylated in TC. High discrimination between TC and thymoma in all 4 genes. No significant differences in the promoter methylation of GNG4, HOXD9, or SALL3 between thymomas and normal thymi. Promoter methylation of the four genes not significantly higher in advanced-stage tumors. Relapse-free survival significantly worse in TEN with a higher DNA methylation of HOXD9 and SALL3.	[50]
GHSR	six type A, six type AB, eight type B1, 11 type B2, 10 type B3 thymomas, 13 TC, four NECTT samples	RT-qPCR, immunohistochemistry	In-1 ghrelin, GHSR1b and GOAT overexpressed in thymoma. No significant differences in the expression of ghrelin and GHSR1a between thymoma and thymic tissue. mRNA expression of In-1 ghrelin and GHSR1b positively associated with GHSR methylation in thymoma tissue samples. No relationship between ghrelin, GHSR1a or GOAT expression and GHSR methylation in thymoma. mRNA expression of GHSR1a and GHSR1b was generally associated with expression of the corresponding protein. Overexpression of GHSR1b in advanced-stage TEN.	[51]
GHSR	12 type A, 12 type AB, five type B1, 22 type B2, eight type B3, five type B2-B3, one not specified thymoma samples	Bisulfite modification, methylation- sensitive high-resolution melting analysis	GHSR almost completely demethylated in the healthy thymic tissues. GHSR hypermethylation observed particularly at stages II-IV than in stage I. No correlation between increased GHSR methylation levels and advancing severity of the MG symptoms. No gender difference observed for GHSR methylation, only a trend for increased methylation with advancing age.	[52]
AIRE	six type A, five type AB, five type B3 thymomas, normal peripheral tissue samples Murine thymi from C57BL/6 mice	Reverse transcriptase quantitative RT-PCR, bisulfite genomic DNA sequencing, quick and quantitative chromatin immunoprecipitation	AIRE promoter hypomethylated in human AIRE-positive medullary and AIRE-negative cortical epithelium. High level of CpG methylation in thymocytes. AIRE promoter uniformly hypomethylated in mouse mTECs. AIRE promoter hypomethylated in AIRE-negative thymomas and peripheral tissues. Positive association of AIRE expression and histone H3 lysine 4 trimethylation in AIRE promoter in human and mouse TEN.	[53]
POMC	three normal thymuses, one large cell lung cancer, five thymic carcinoid tumors	Immunohistochemistry, quantitation of POMC gene expression, bisulphite modification and sequencing	Hypermethylation in the 5-promoter region of the POMC gene in normal thymuses and large cell lung cancer. Hypomethylation in TC tumors with ectopic ACTH syndrome. Hypermethylated region narrowed to coordinates −417 to −260 of the POMC promoter. Levels of POMC expression correlated with the methylation density at –417 to –260 bp across the E2 transcription factor binding region of the POMC promoter.	[54]
MTHFR, DNMT3A	13 type A, 13 type AB, five type B1, 23 type B2, eight type B3, five type B2/3, two non-specified thymoma samples	Bisulfite modification, methylation- sensitive–high-resolution melting analysis	Hypermethylation of both MTHFR and DNMT3A promoters in TEN. Both DNMT1 and DNMT3B promoter regions mostly hypomethylated in all investigated tissues. MTHFR methylation increased in thymomatous MG.	[55]
PTEN	three type A, eight type AB, 11 type B1, six type B2, five type B3 thymoma samples, four TC, two normal thymi	Immunohistochemistry, PCR direct sequencing, methylation-specific PCR, reverse transcription real-time PCR after target cell collection with laser microdissection	PTEN protein expressed in type A thymoma and TC cells. Neither PTEN mutations nor promoter methylation detected in any samples. PTEN mRNA expression lowest in type A thymoma cells.	[56]
Dlc1	Thymic cell culture Transgenic mice	Fluorescence microscopy, multiplex RT-PCR, western blotting, immunohistochemistry, microdissection, loss of heterozygosity analysis, DNA methylation study of Dlc1 promoter region, transendothelial migration and in vitro filipodia formation assay of the T lymphoma cells, treatment of cell culture with 5 azacytidine	Significantly shortened life spans in mice heterozygous for the gt Dlc1 allele and an inducible LSL-K-Ras2G12D allele compared with the LSL-K-Ras2G12D only mice. High degree of lung metastasis in heterozygous mice. Tumor specific selective hypermethylation of the Dlc1 isoform 2 promoter and reduction of the corresponding protein expression in thymic lymphoma and TC. Increased trans-endothelial cell migration in Dlc1 deficient thymic lymphoma cell lines. Increased stress fiber formation and Rho activity in TC cell lines. Different morphological changes after introduction of the three Dlc1 isoforms tagged with GFP into these cells.	[57]
TET2	nine squamous cell carcinomas, one undifferentiated carcinoma	Exome sequencing, analysis of mutational signature, genome copy number analysis and tumor content estimation, whole transcriptome sequencing, DNA methylation analysis, gene ontology analysis	Recurrent somatic mutations in TET2, CYLD, SETD2, TP53, FBXW7, HRAS and RB1, and no mutations in GTF2I. More hypermethylated genes in TC with TET2 mutations. Hypermethylation associated with down-regulation of gene expression. Elevated gene expression at the KIT and AHNAK2 gene loci.	[58]
GAD1	nine type A, 11 type AB, 19 type B1, 20 type B2, 14 type B3, 17 type C thymoma samples	Bisulfite conversion of genomic DNA, global methylation analysis, bisulfite pyrosequencing, RT-qPCR, immunohistochemistry	Hypermethylation and significantly higher levels of mRNA and protein expression of GAD1 in TC. Correlation between high GAD1 levels and significantly shorter relapse-free survival rates. High sensitivity and specificity of GAD1 methylation for the discrimination between TC and thymoma.	[59]
KITLG	121 thymoma samples from TCGA-THYM dataset, 37 samples from GEO dataset-GSE29695 Thy0517 cell line	RNA-seq data analysis, KITLG small-interfering RNA silencing, KITLG-overexpressing plasmid construction, real-time PCR, western blot analysis	KITLG overexpression in type A and AB thymoma. 220 up- and 72 down-regulated genes at the mRNA level, 79 positive and 78 negative miRNAs, 28 hypermethylation and 163 hypomethylation regions through high expression of KITLG. GRB2 expression and phosphorylation levels of BRAF, MEK1/2, and ERK1/2 in the MAPK pathway positively correlated with the change in KITLG in Thy0517 cells.	[60]

**Table 3:** Alterations of histone modifications in TENs.

HDACI	Cell Lines/Tissue Samples/Patient Collective	Methods	Main Results	References
Belinostat, Panobinostat	Bronchial metastatic lesion of a 54-year old female patient with a metastatic thymoma with pleural invasion	Mutation analysis, high content imaging drug screening	High efficacy on the cytokeratin-19+ and lower efficacy on the vimentin+ cells.	[61]
Belinostat	25 patients with thymoma, 16 patients with TC	Pharmacodynamic analyses: Protein acetylation, peripheral blood mononuclear cell immune subsets, circulating angiogenic markers	Two patients with thymoma had partial response, 25 stable, and 13 progressive disease. No responses among patients with TC. Significantly longer survival of patients with thymoma. Protein acetylation, regulatory T-cell numbers, and circulating angiogenic factors did not predict the outcome.	[62]
Belinostat	two patients with type B1, seven with type B2, three with type B3 thymoma, 14 patients with TC	Monitoring for treatment-related adverse events, clinical laboratory tests, vital signs, physical examinations, 12-lead electrocardiograms, pharmacokinetic evaluations, multiparametric flow cytometry, CT scan	Objective response rates of 64% in thymoma and 21% in TC. Modulation of pharmacodynamic markers of HDAC-inhibition and declines in regulatory T cell and exhausted CD8+ T cell populations. Decline in regulatory T cell affected response and progression-free survival. Declines in TIM-3+ CD8+T cells greater in responders.	[63]
VPA	10 peritumoral thymi, 14 thymomas, 5 TC	Microarray hybridization, RT-qPCR, immunohistochemistry, transfection and treatment, lysate preparation and immunoblotting analysis, transwell migration assay, clonogenic assays, immunocytochemistry and morphological analysis, cell cycle analysis, ATPlite luminescence assay system, formaldehyde cross-linking and chromatin immunoprecipitation, plasmid construction and dual-luciferase reporter assay	HDAC inhibition promoted miRNA-145-5p expression, reduced tumor phenotype, and sensitized TEN cells to chemotherapy and epidermal growth factor receptor (EGFR) tyrosine kinase inhibitor. Up-regulation of miRNA-145-5p and concomitant down-regulation of miRNA-145-5p target genes after VPA treatment of TC1889 cells. Antitumor effects of VPA, as indicated by the induction of cell cycle arrest and by the reduction of cell viability, colony forming ability and migration capability. Reduction of VPA treatment impact on cell viability and colony forming ability of TEN cells through hampering of the miRNA-145-5p activity by a LNA inhibitor. VPA enhanced TEN cell response to cisplatin and erlotinib.	[64]

## Data Availability

Not applicable.
